# Lung cell toxicological effects of 3D printer aerosolized filament byproducts

**DOI:** 10.1007/s11356-025-36006-1

**Published:** 2025-02-04

**Authors:** Jonathan M. Beard, Brooke M. Royer, Jacob M. Hesita, Peter Byrley, Ashley Lewis, John Hadynski, Joanna Matheson, Souhail R. Al-Abed, Christie M. Sayes

**Affiliations:** 1 Department of Biology, Baylor University, Waco, TX 76798 USA; 2 Department of Environmental Science, Baylor University, One Bear Place #97266, Waco, TX 76798-7266 USA; 3 Health and Environmental Effects Assessment Division, U.S. EPA, Research Triangle Park, NC 27711 USA; 4 Oak Ridge Institute of Science and Education (ORISE) research participant to the U.S. EPA, Research Triangle Park, NC 27711 USA; 5 Center for Environmental Solutions and Emergency Response, U.S. Environmental Protection Agency, 26 W. Martin Luther King Dr, Cincinnati, OH 45268 USA; 6 Office of Hazard Identification and Reduction, U.S. Consumer Product Safety Commission (CPSC), 4330 East-West Highway, Bethesda, MD 20814 USA

**Keywords:** Particulate matter, PLA, ABS, Glutathione, Cytotoxicity, Volatile organic compounds

## Abstract

As 3D printing has become more compact and affordable, the use of the technology has become more prevalent across household, classroom, and small business settings. The emissions of fused filament fabrication (FFF) 3D printers consist of volatile organic compounds (VOCs) and aerosolized particulate matter (PM) dependent upon the filament in use. This study investigates the hazards posed by these emissions through aerosol characterization and cell exposure. Seventeen filaments were obtained from five manufacturers, consisting of fourteen plastic filaments (polylactic acid (PLA), acrylonitrile butadiene styrene (ABS), or polycarbonate (PC) polymers) and three filaments with metal filler (copper, bronze, and steel). For 1-h trials, BEAS-2B human bronchial epithelial cells were directly exposed to aerosolized 3D printer emissions at the air–liquid interface (ALI). Particle characterization showed ABS filaments produced more PM and VOC emissions with particles in the ultrafine size range. ABS filaments also elicited a greater biological response, with significant shifts in mitochondrial activity compared to the PLA filaments. Significant changes in amounts of glutathione (GSH) were observed after ABS and PLA emission exposure. Exposure to emissions from the steel filament resulted in the lowest average amount of glutathione, though insignificant, and a significantly lower mitochondrial activity, revealing a unique cause for concern among filaments tested. 3D printer emissions and subsequent cell responses appear filament-dependent, and users should mitigate personal exposure to aerosols.

**Graphical abstract:**

Made using elements from BioRender.

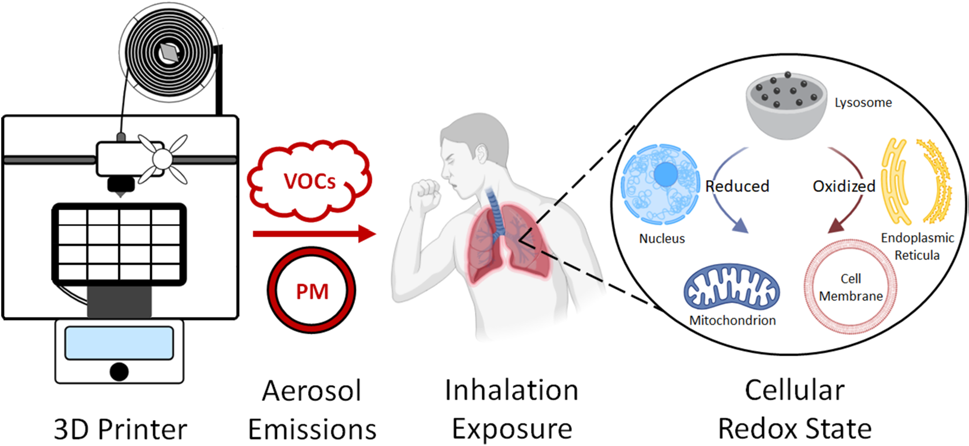

## Introduction

Additive manufacturing, colloquially referred to as “3D printing,” is the technology in which three-dimensional objects can be built layer-by-layer by an automated machine (printer) using a computer-aided design (CAD) file (Shahrubudin et al. 2019). 3D printing can be categorized into discrete types, such as material extrusion (*e.g.,* fused filament fabrication), vat photo-polymerization (*e.g.,* stereolithography), and powder bed fusion (*e.g.,* selective laser sintering) (Alfattni 2022). Today, fused filament fabrication (FFF) is the most popular 3D printing category, first patented in 1989 (US5121329A 1989). This printing style has resulted in an explosion in 3D printing due to low costs and ease of use (Ćwikła et al. 2017; Jain & Jain 2021; Li et al. 2017). FFF directly extrudes filaments onto the build plate by heating them to high temperatures in the nozzle, either melting or softening the material to a malleable state. Desired filaments are composed of thermoplastics such as polylactic acid (PLA), acrylonitrile butadiene styrene (ABS), or polycarbonates (PC). Filaments can also comprise ceramics, wood, glass, metals, and metal composites (Ngo et al. 2018; Ranjan et al. 2022; Zhang et al. 2021).

When filaments are extruded, aerosolized particulate matter (PM) and gaseous volatile organic compounds (VOCs) are produced (Azimi et al. 2016; Byrley et al. 2020; Gu et al. 2019; Zhang et al. 2019). PM is known to adversely affect cardiovascular health and respiratory health and has led to increased hospitalizations and deaths (Bartoli et al. 2009; Brauer et al. 2012; Guaita et al. 2011; Halonen et al. 2009; Kim et al. 2015; Mills et al. 2009; Russell and Brunekreef 2009). The mode of toxicity of aerosols is partially influenced by the primary particle size (measured in aerodynamic diameter), whereby smaller particles can penetrate deeper into the pulmonary region and have the potential to translocate from the lungs to the circulatory system (Brown et al. 2013; Hamanaka and Mutlu 2018). Three distinct size classes for particulate matter exist: coarse particulate matter (10 µm or smaller, PM_10_), fine particulate matter (2.5 µm or smaller, PM_2.5_), and ultrafine particulate matter (100 nm or smaller, PM_0.1_) (Kim et al. 2015). Differences in physicochemical properties such as size and chemical composition influence toxicological mechanisms, producing distinct health effects such as cancers, neurodegenerative disorders, and cardiopulmonary diseases (Shah et al. 2013; Shou et al. 2019; Thangavel et al. 2022).

VOCs are byproducts of natural plant metabolic processes and various anthropogenic activities, including energy production and manufacturing (Gu et al. 2021; Michanowicz et al. 2022; Rissanen 2021; Zhou et al. 2023). The toxicity of indoor VOCs can be assessed by chemical class and concentration (Gostner et al. 2016). Compounds with higher volatility enter the gas phase more readily, posing environmental and health hazards at lower exposure levels than less volatile compounds (David and Niculescu 2021; Gostner et al. 2016). Indoor VOCs have been linked with respiratory irritation, asthma, aggravation of allergies, and “sick-building syndrome” (Alford and Kumar 2021; Gostner et al. 2016; Sahlberg et al. 2013; Wolkoff et al. 2006).

Redox equilibrium, the balance between oxidized and reduced states of chemical messengers, cofactors, enzymes, and organelles, produces an intricate system of localized oxidation states necessary for cellular homeostasis (Sies et al. 2017). The disruption of this balance in either direction can result in biological damage through oxidative or reductive stress. Glutathione (GSH) quantity can be used as a cellular marker of redox stress. GSH is converted into glutathione disulfide (GSSG) at a higher rate during cellular oxidative stress, resulting in more intracellular GSSG and less GSH (Jones 2006; Mytilineou et al. 2002). However, both GSH and GSSG concentrations have increased in some human lung conditions and during oxidative stress in mung beans (Rahman and MacNee 2000; Yu et al. 2002). Furthermore, changes in GSH levels can occur quickly within a cell within 5 min of exposure to hydrogen peroxide (Hohnholt and Dringen 2014; Jiang et al. 2017). Disrupting the redox equilibrium through 3D printing emissions would interfere with the vital metabolic processes throughout the cell, and this disruption could be assessed by quantifying GSH.

The present study characterizes particle and gaseous emissions from 3D printers and analyzes their downstream biological effects across seventeen unique 3D printing filaments. Some studies have investigated the physicochemical characteristics of 3D printer emissions without probing cellular effects (Chan et al. 2020; Zontek et al. 2017), and the current studies that do examine in vitro effects often consist of only three or fewer total filament treatments (Fang et al. 2023; Farcas et al. 2022; He et al. 2024; Kim et al. 2022). Our analysis greatly expands this typical sample size by using many ABS- and PLA-based filaments to investigate tangential variables such as brand, color, and additives that could confound abstracted conclusions regarding the effect of these polymer types on human health and safety. After characterizing the aerosols emitted by each filament, the effect of the total emissions on cell health was determined using mitochondrial activity (as measured by the MTS assay) and oxidative stress markers (as measured by the GSH-Glo assay). These cellular assays were performed after using a novel exposure method for 3D printing hazard assessment. A self-contained aerosol exposure chamber (CelTox Sampler) was used to expose cells at the air–liquid interface (ALI) for 1-h trials. The chamber enhances deposition while minimizing cell death and stress by maintaining constant temperature (37 °C) and humidity during exposure. The exposure period was selected as a realistic simulation of classroom and household 3D printing exposure scenarios.

## Materials and methods

### Materials

Nunc™ EasYFlask™ 225 cm^2^ (T225) cell culture flasks, Corning™ Costar™ six-well clear tissue culture-treated flat bottom plates, Falcon™ 50 mL conical centrifuge tubes, Basix™ microcentrifuge tubes, Invitrogen™ Countess™ cell counting chamber slides, Nunc™ 25 mL serological pipettes, Greiner Bio-One CELLSTAR 24-well cell culture plates, Corning™ 96-well solid white non-sterile microplates, 9″ and 5 ¾” glass Pasteur pipets, Gibco™ trypsin–EDTA (0.25%) phenol red, Gibco™ DMEM/F-12 with l-glutamine and phenol red, Corning™ penicillin–streptomycin (pen-strep) solution (10,000 IU penicillin, 10,000 µg/mL streptomycin), and Corning™ regular fetal bovine serum (FBS) were purchased from Fisher Scientific (Pittsburgh, PA, USA). Translucent, high-density, 0.4 µm inserts for six-well plates were purchased from VWR International (Radnor, PA, USA). Silicone tubing and pipe fitting adapters were purchased from Amazon (Seattle, WA, USA). BEAS-2B human bronchial epithelial cells were purchased from ATCC (Manassas, VA, USA). CelTox Sampler and six-well aluminum carrier plates were provided by MedTec Biolab (Durham, NC, USA). Contemporary FFF 3D printer and 3D printing filaments were provided by the US Environmental Protection Agency (EPA) (Washington, D.C., USA).

### Cell culture conditions

BEAS-2B cells were used between passage numbers 11 to 30. Cells were grown in T225 flasks at standard conditions (37 °C, 5% CO_2_, water-saturated atmosphere) with DMEM media containing 10% FBS and 1% pen-strep solution. At 90–100% confluency, cells were plated on six-well inserts at 300,000, 150,000, or 75,000 cells per well and exposed two, three, or four days later, respectively. Apical media was removed one day before exposure to acclimate cells to the ALI. Cells were counted using an Invitrogen™ Countess™ 3 FL automated cell counter, stored in a Fisherbrand Isotemp CO_2_ Incubator, and spun down in an Eppendorf™ 5810 R benchtop centrifuge at 300 RCF for 5 min.

### Exposure to 3D printing emissions

**Fig. 1:**
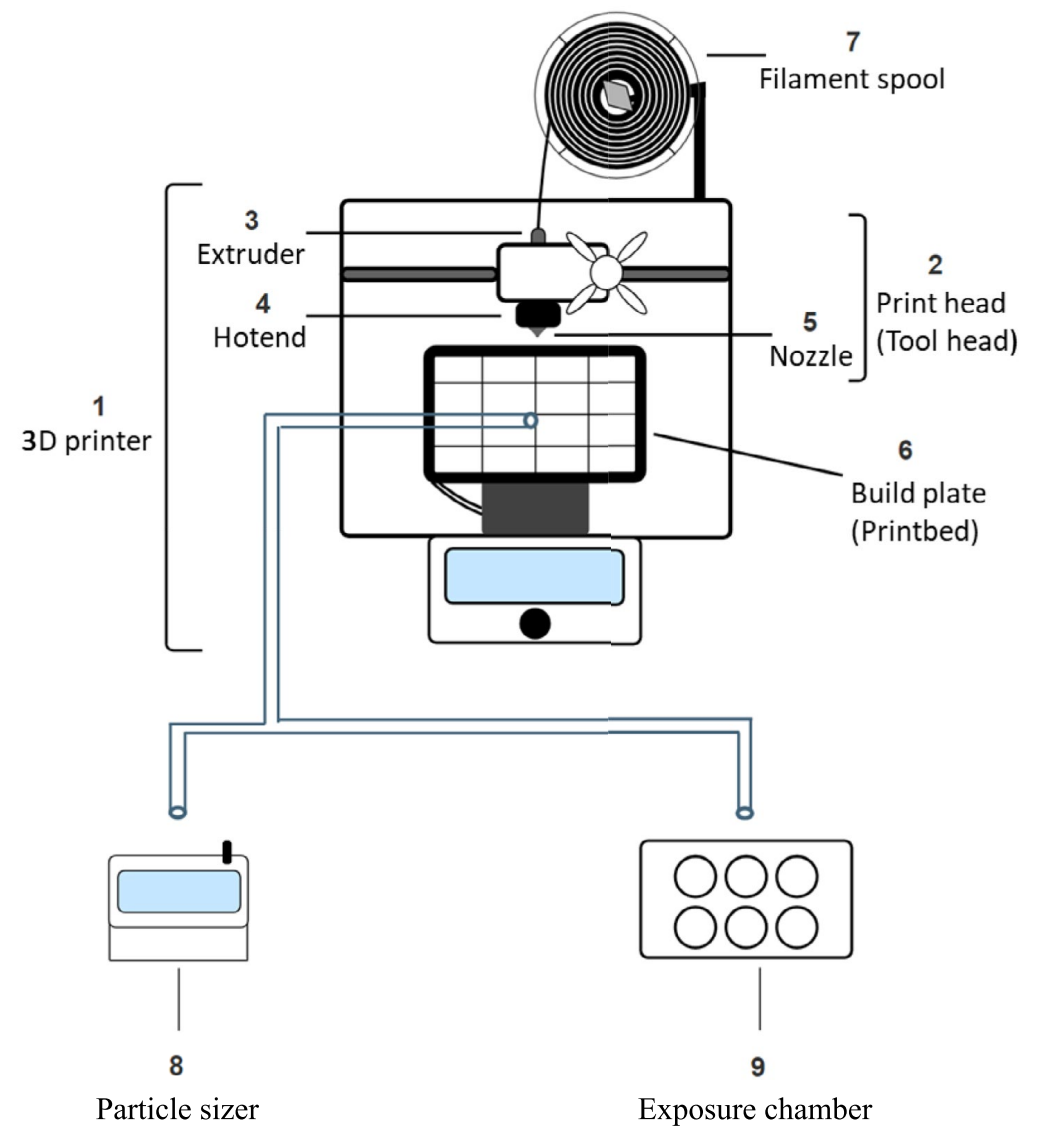
Schematic depicting direct exposure of cells within the CelTox Sampler exposure system to total emissions from the 3D printer. Insets include (1) 3D printer, (2) print head/tool head, (3) extruder, (4) hotend, (5) nozzle, (6) build plate/printbed, (7) filament spool, (8) particle sizer, and (9) exposure chamber. Test air was pumped to the particle sizer or exposure chamber through a single tube

Cells were exposed to emissions from seventeen unique filaments at the ALI. The treatments include seven ABS filaments, six PLA or PLA + filaments, and four “Other” filaments including steel, bronze, copper, and polycarbonate (ESD-PC). The metal filaments are PLA-based filaments with a metal powder filler and were described by the online vendor as containing 80% of their respective metal components. The filaments’ details, such as type, color, and operational temperature, are reported (Table 1). Filaments were printed using a desktop FFF 3D printer with an M175 v2 1.75 mm tool head. A plexiglass case surrounded the 3D printer during exposures to minimize environmental emission loss. Each exposure began after the initial layer was printed and lasted 60 min. Whole 3D printer emissions were pumped through silicone tubing immediately before the nozzle directly onto cells in the CelTox Sampler exposure chamber. Six-well inserts containing cells were transferred from Corning™ culture-treated plates to MedTec aluminum carrier plates preheated to 37 °C with 1 mL of media in each well before exposure. Cells were kept at 37 °C within the exposure chamber, and incoming air was humidified before being deposited onto cells. After exposure, cells were returned to Corning™ culture-treated plates with either fresh media or trypsin in preparation for biological assays. The bespoke exposure setup is illustrated (Fig. 1).

**Table 1:** List of filaments used in this study, including the descriptive characteristics type, brand, color, diameter, net weight, and the temperature settings of the build plate and hotend during print

Type	Brand	Color	Diameter (mm)	Net weight (kg)	Build plate temp (°C)	Hotend temp (°C)
ABS	A	Black	1.75	1.00	110	240
ABS	B	Black	1.75	1.00	110	240
ABS	B	Green	1.75	1.00	110	240
ABS	C	Black	1.75	1.00	110	240
ABS	C	Green	1.75	1.00	110	240
ABS	D	Blue	1.75	1.00	110	240
ABS	D	Green	1.75	1.00	110	240
PLA	B	Green	1.75	1.00	60	195
PLA	C	Black	1.75	1.00	60	195
PLA	C	Blue	1.75	1.00	60	195
PLA	C	Green	1.75	1.00	60	195
PLA +	B	Black	1.75	1.00	60	195
PLA +	B	Blue	1.75	1.00	60	195
Bronze	E	N/A	1.75	0.75	60	195
Copper	E	N/A	1.75	0.75	60	195
Steel	E	N/A	1.75	0.75	60	195
ESD-PC	A	Black	1.75	0.75	110	275

Acronyms: acrylonitrile butadiene styrene (ABS), polylactic acid (PLA), polylactic acid plus (PLA +), and electrostatic discharge polycarbonate (ESD-PC). Metal types are PLA-based with partial metal filler

### Particle characterization

Particulate matter emissions for each filament were measured using an Optical Particle Sizer 3330 (OPS) and a NanoScan Scanning Mobility Particle Sizer 3910 (SMPS™) from TSI (Shoreview, MN, USA) in particles/cm^3^. OPS measured particles ranging from 300 nm to 10 µm, and SMPS™ measured particles from 10 to 300 nm. Scans lasted for 60 s and were started after the initial layer was printed. Scans were conducted in a biosafety cabinet to avoid background readings from general particulate matter in the air. Particle data was converted to continuous data following TSI’s instructions and visualized in RStudio 2022 12.0 using ggplot2, viridis, scales, and ggpubr packages. Assumptions of constant density (1 g/cm^3^) and shape for particulate matter were required to convert from discrete to continuous and produce mass data and dN was normalized to dN* to compare distributions agnostic of total counts.

### VOCs characterization

Total VOCs were examined using a simulated FFF method consisting of an Agilent 8890 GC with 7010B GC/TQ MS equipped with Gerstel multipurpose sampler (MPS), cryotrap, and thermal desorption unit (TDU 2.0). For each filament, a quartz thermal desorption tube was packed with a bed of glass wool and then loaded with ~ 1/2″ pieces of 3D printing filament until about 10–75 mg of filament was in the tube and was then covered with a second bed of glass wool. The filament mass varied in correlation to density. The filaments were then heated in the TDU with a vent time of 3.33 min at 50 °C to remove any water absorbed in the sample from ambient humidity, followed by the sample heating for 3 min at a temperature simulating recommended printhead temperatures for the given filament (ABS: 240 °C, PLA: 210 °C, ESD-PC: 300 °C). The cryotrap concentrated the emitted compounds and injected them into an Agilent DB-624 UI column. The chromatograms were deconvoluted using Agilent Masshunter (Santa Clara, CA, USA).

### Mitochondrial activity assay (MTS)

Mitochondrial activity was measured using the MTS [3-(4,5-dimethylthiazol-2-yl)−5-(3-carboxymethoxyphenyl)−2-(4-sulfophenyl)−2H-tetrazolium] assay. This is a colorimetric assay in which MTS tetrazolium is reduced by cells into a colored formazan product. The amount of formazan produced depends upon the number of living cells and the overall mitochondrial activity. This assay was run per the manufacturer’s instructions (CellTiter 96® AQ_ueos_ One Solution Cell Proliferation Assay, Promega, Madison, WI, USA) 24 h post-exposure with a 2-h incubation time. Cell media was replaced immediately after exposure and again immediately before the MTS assay. One milliliter of media was added to the apical and basal side of the insert with 0.2 mL of MTS solution. After incubation, apical and basal solutions were combined and moved to 24-well plates to be scanned in triplicate at 490 nm absorbance on a Synergy H1 microplate reader from BioTek (Winooski, VT, USA).

### Glutathione assay

Oxidative stress was probed using the GSH-Glo Glutathione assay (Promega). This assay produces a signal measured in luminescence that is proportional to the amount of GSH present. GSH is measured through a combined reaction using the enzyme glutathione S-transferase and the luciferase enzyme used for bioluminescence in fireflies and similar beetles, whereby a higher quantity of GSH results in a greater conversion of a luciferin derivative (Luc-NT) into luciferin, which luciferase then utilizes to produce light. The assay was performed per the manufacturer’s instructions with cells treated as mammalian cells in suspension. Cells were trypsinized immediately after exposure to detach cells from the insert with 0.9 mL of trypsin on both the apical and basal side of the insert. After an 8-min incubation, apical trypsin was collected, and the inserts were washed with 1 mL of media. The collected cell solution was spun down at 300 RCF for 5 min, the supernatant aspirated, and the cell pellets resuspended in 170 µL of dH_2_O or phosphate-buffered saline to avoid phenol red interference. The cell solution (10 µL) was used for assessing cell counts, and 50 µL of cell solution was plated in triplicate on a 96-well white plate. Luminescence was measured on a Synergy H1 microplate reader from BioTek (Winooski, VT, USA) with a 1-s integration time. Net RLU was calculated by subtracting the blank well values from the cell-containing wells. The net RLU value was then used during normalization to produce the final GSH values.

### Statistics, controls, and data visualization

Data for GSH and MTS are expressed as normalized mean ± standard deviation, where *n* = number of biological replicates. All GSH and MTS data were normalized to negative control (untreated) groups from the same plating group, including experimental treatments and vehicle controls. Experimental treatments were statistically compared to the vehicle control, which was exposed to laboratory air as a sham treatment. Untreated cell groups were plated alongside treatment groups and were transitioned to ALI but were never transferred to aluminum plates or moved to the CelTox sampler. Vehicle control groups were further transitioned to aluminum plates and moved to the CelTox sampler for an equal-duration exposure to regular laboratory air instead of 3D printer emissions.

The normality of the data was tested using a graphical test by standardizing the data using the average and standard deviation, sorting the data into bins, and graphing a histogram of the frequency of the standardized deviation in each bin. If the data was normal, a single-factor ANOVA was run to determine if there was a significant difference between any of the treatments. If there was a significant difference according to the ANOVA (*p* < 0.05), a *t*-test was used to determine if each treatment differed significantly from the control. Before performing the *t*-test, an *F*-test was utilized to determine if variances were unequal. If the *F*-test was significant, Welch’s *t*-test was used to account for the heterogeneity in the variances. For both GSH and MTS data sets, the test of normality and *F*-test bore the same result; thus, the same ANOVA and *t*-test were used for both data sets. An outlier was only removed at any level of analysis if it was significantly different according to a *Q*-test. A *p*-value < 0.05 was considered significant, < 0.01 highly significant, and < 0.001 very highly significant.

## Results

### Characterization of particulate matter and VOCs

**Fig. 2:**
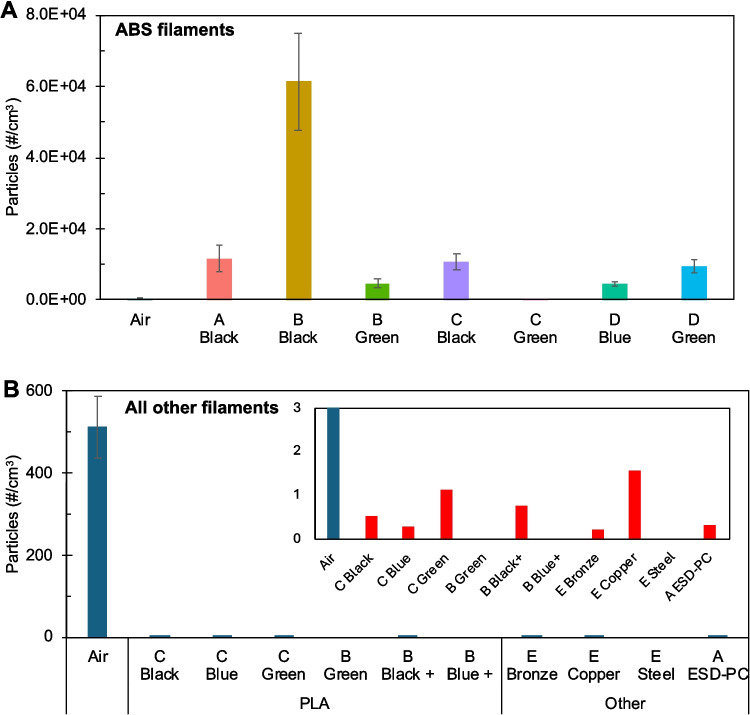
Number of particles emitted as measured with SMPS and OPS. Data represented as #/cm^3^ of aerosolized particles emitted by **A** ABS filaments and **B** all other filaments with laboratory air as a control. The inset in panel **B** shows the same data with a magnified y-axis

**Fig. 3:**
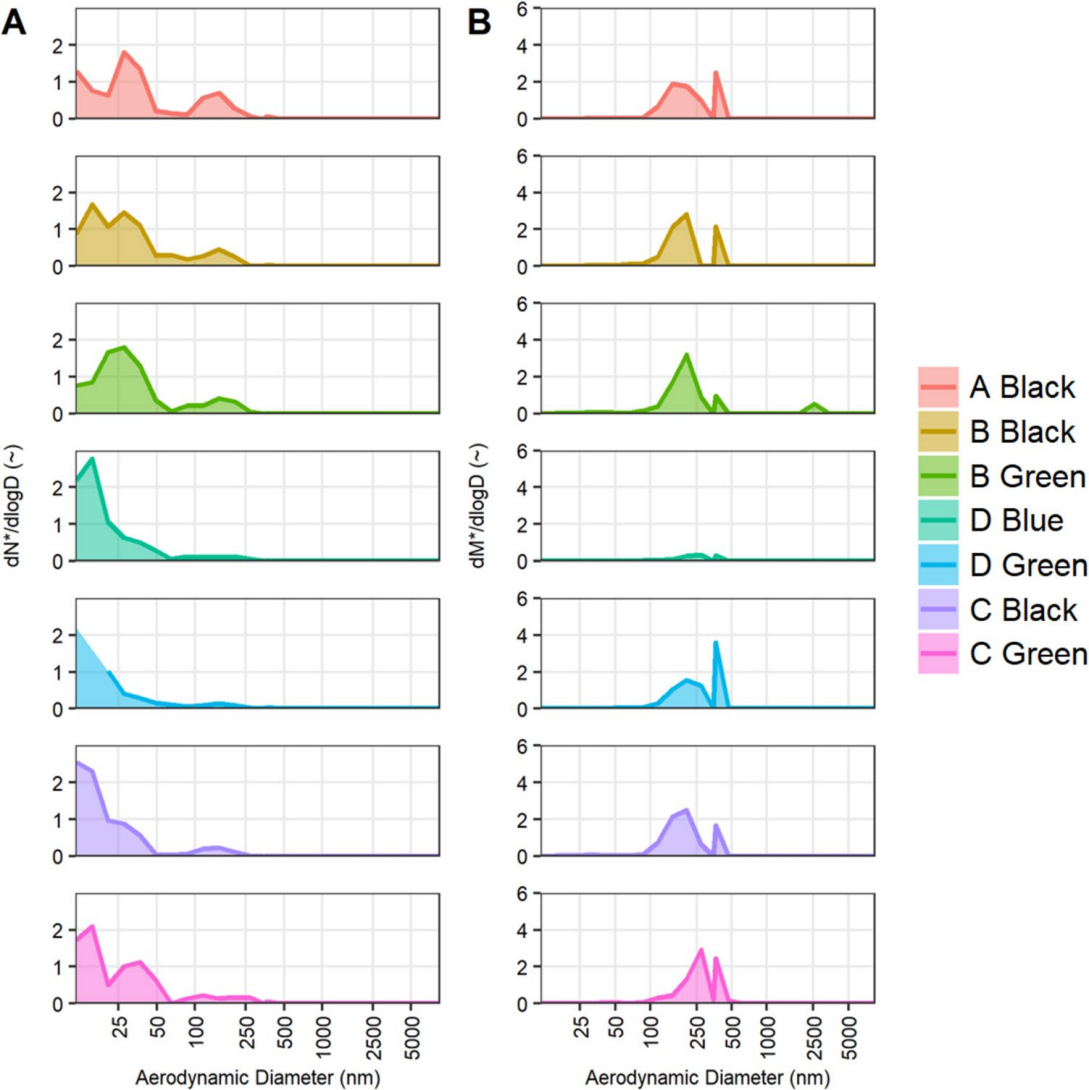
Characterization of aerosolized particles produced by ABS polymer filaments. **A** Number of particles [dN*/dlogD] and **B** mass of particles [dM*/dlogD] across aerodynamic diameter. Raw count was normalized to proportion of total to compare distributions agnostic of total count

The total number of particles (#/cm^3^) emitted by each filament was measured using an average of 60 s scans with an OPS and SMPS (Fig. 2A, B). The number of particles in the air outside the biosafety cabinet was also measured as a baseline for comparison. All ABS filaments emitted more particles than the air control except the Brand-C green ABS filament (Fig. 2A). The Brand-B black ABS filament emitted the highest particle count, amounting to over 6.1 × 10^4^ particles/cm^3^. The Brand-B green ABS filament emitted the fewest particles that surpassed the air control with 4.6 × 10^3^ particles/cm^3^. On the other hand, all PLA, bronze, copper, steel, and ESD-PC particle emissions did not exceed air control levels (Fig. 2B). The total number of particles in Fig. 2 is the combined data from the SMPS and OPS readings; however, almost the entire total was detected using SMPS. This discrepancy in particle size is further shown in Fig. 3.

Particle aerodynamic size and mass data were calculated for the ABS filaments with emissions that surpassed air control levels (Fig. 3A, B). The size and mass distributions are represented by log distributions dN*/dlogD and dM*/dlogD, respectively. The results indicate that most of the particles emitted by ABS filaments were under 50 nm in diameter (Fig. 3A). Very few, if any, of the particles had an aerodynamic diameter greater than 500 nm. Due to the nature of the mass-to-size ratio, even the few particles in the 100–500 size range comprised most of the mass distribution (Fig. 3B).

**Fig. 4:**
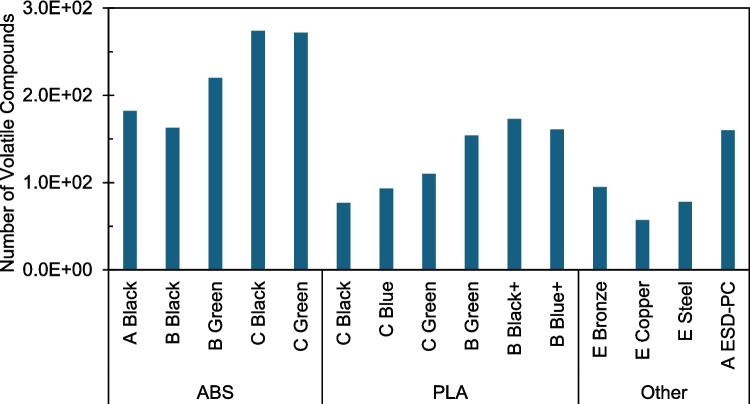
Number of volatile organic compounds produced from each filament during simulated fused filament fabrication (FFF) using a thermal desorption unit

The total number of VOCs for each filament was determined using a simulated FFF approach utilizing a thermal desorption unit to heat filament pieces at temperatures emulating 3D printer hotends (Fig. 4). This method showed that ABS filaments generally produced more total VOCs than the PLA and PC filaments tested, including PLA filaments with metal additives. The number of VOCs detected for Brand-C ABS filaments was generally higher than those of other ABS brands. In contrast, Brand-B PLA filaments produced a higher number of VOCs than Brand-C PLA filaments. In this approach, metal additives (copper, bronze, and steel; Brand E) appeared to have little effect on the number of VOCs emitted and, like Brand-C PLA filaments, were lower than Brand-B PLA filaments.

### 3D printer emission effects on cell viability and GSH

**Fig. 5:**
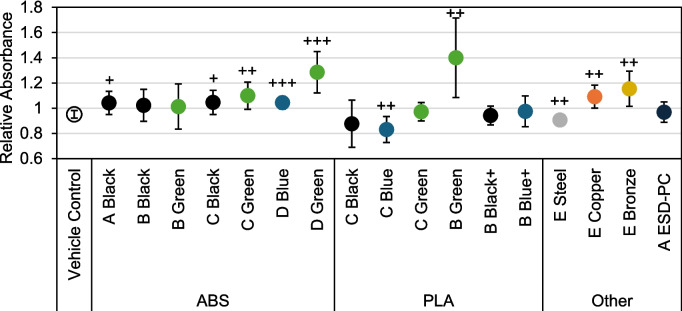
MTS results for all filaments exposed to BEAS-2B cells. Absorbance was normalized to untreated cells from the same passage as each treatment. Filaments were statistically compared to vehicle control via a two-tailed, two-sample heterogeneous variance *t*-test. +  = *p* < 0.05. +  +  = *p* < 0.01. +  +  +  = *p* < 0.001. *N* = 9 for all filaments

The metabolic activity of BEAS-2B cells was recorded using the MTS assay after exposure to 3D printing filaments (Fig. 5). Activity was measured in relative absorbance units (RAU), and the absorbance value of each treatment group and vehicle control was normalized to the untreated cell average. The average of each filament group was compared to the average of the vehicle control using Welch’s *t*-test. Most ABS filaments induced a significant increase in metabolic activity compared to the control (Brand-A black (*p* < 0.05), Brand-C black (*p* < 0.05), Brand-C green (*p* < 0.01), Brand-D blue (*p* < 0.001), and Brand-D green (*p* < 0.001)). In the PLA filament group, Brand-B green was significantly higher than the control (*p* < 0.01), and Brand-C blue was significantly lower than the control (*p* < 0.01). Cell activity varied with metal-based filament exposure. Steel was significantly lower (*p* < 0.01) than the air control, while bronze and copper were significantly higher (*p* < 0.01). Brand-B green PLA emissions caused the highest average activity at 1.40 normalized RAU, and Brand-C blue PLA resulted in the lowest at 0.830 RAU.

**Fig. 6:**
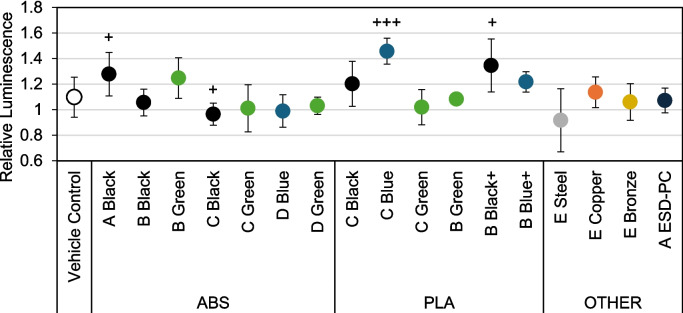
Total GSH for all filaments exposed to BEAS-2B cells. Luminescence normalized to untreated cell average from the same passage as each treatment. Filaments were statistically compared to vehicle control (i.e., cells exposed to air alone in the same exposure conditions) via a two-tailed, two-sample heterogeneous variance *t*-test. +  = *p* < 0.05. +  +  +  = *p* < 0.001. *N* = 9 for all filaments

The amount of GSH was recorded immediately after the 1-h exposures of BEAS-2B cells to emissions from each filament (Fig. 6). GSH was measured in relative luminescence units (RLU), which describe luminescence normalized to the untreated control after subtracting the negative control (blank wells). After normalizing the data, each filament was compared to the vehicle control using Welch’s *t*-test. Only the Brand-A black ABS (*p* < 0.05), Brand-C blue PLA (*p* < 0.001), and Brand-B black PLA + (*p* < 0.01) filaments were associated with a significant increase in total GSH at 128%, 146%, and 135% of the untreated, respectively. In contrast, Brand-C black ABS caused a significant decrease in total GSH at 96% of the untreated (*p* < 0.05). While steel was not significant, it did produce the lowest average GSH at 91.7% of the untreated compared to the vehicle control’s 109% total GSH.

## Discussion

This study aimed to thoroughly analyze a wide range of 3D printer filaments to assess the potential toxicological impact after aerosol exposure at the air–liquid interface. The hazard assessment was pursued through a combined physicochemical characterization of the aerosol emissions and a cellular post-exposure analysis. The cell model used in this study was BEAS-2B bronchial epithelial cells. BEAS-2Bs were selected as a model of pulmonary health since the bronchial epithelium comprises the first line of defense in the lungs (Holgate et al. 1999; Newhouse et al. 1976; Sibille and Reynolds 1990). The characterization of the aerosols showed that ABS filaments produced more PM than PLA or other filaments, specifically in the ultrafine size range (< 100 nm). Similarly, ABS filaments produced a greater effect on the cells than PLA filaments, particularly in the MTS assay. While these filaments did not produce a lower shift in metabolic response indicative of cell death, more significantly different filaments in the ABS category showed a heightened metabolic response, possibly indicating increased cell stress due to exposure to ABS-based printing emissions. The steel filament produced low results in both assays, showing a significant reduction in metabolic activity (*p* < 0.01) and the lowest average GSH response. This could indicate general cytotoxicity after exposure to steel filament 3D printing emissions and cytotoxicity specifically due to oxidative stress.

The total particle emissions from ABS filaments were consistently higher than those from PLA or other filaments, including metal-filler and PC filaments. According to the WHO, an acceptable level of nanoparticles is 20,000 or fewer particles/cm^3^ across a 1-h average (WHO 2021). The filament with the greatest number of particle emissions, ABS black (Brand-B), had nearly triple that amount across a 60 s sweep. The contrasting trend in particle emission between ABS and PLA filaments is consistent with other findings in the literature, although inconsistencies depend on the individual filament (Khaki et al. 2021; Tang and Seeger 2022; Tedla and Rogers 2023; Yi et al. 2016). Additives and undeclared impurities can have a strong influence on emissions (Hill et al. 2022; Potter et al. 2021; Tang and Seeger 2022; Zhang and Black 2023). Due to the potential for a large number of emitted particles, operators of 3D printer with ABS filaments are recommended to either reduce their overall exposure time or confine the 3D printer to spaces with an effective extraction system (Garcia-Gonzalez et al. 2024). The total number of particles detected is not a direct representation of the number of particles exposed to the cells but instead was used to compare the relative output from the different filaments. This is due to two factors regarding particle characterization methodology: shorter aerosolization duration (60 s instead of 60 min) and location within a biosafety cabinet with constant background air circulation. All filaments likely produced more particles during cell exposures, which were performed under more realistic conditions than the measurements taken in the biosafety cabinet.

The size range distribution of particulate matter could only be calculated for ABS filaments, which produced a sufficiently high number of particles for further analysis. The size range detected here, spanning 10 to 250 nm, is reasonable according to studies in which 3D filaments released particles in the ultrafine size range (Byrley et al. 2019; Romanowski et al. 2023; Sittichompoo et al. 2020; Tang and Seeger 2022; Yi et al. 2016). This indicates the majority of PM released by 3D printing is in the ultrafine size range, allowing the particles to not only deposit throughout the lungs and pharyngolaryngeal region but permeate into the bloodstream and be distributed throughout various tissues throughout the body. Particle mass distribution was also as expected, with larger particles making up most of the distribution. The mass and number distributions exhibit a bimodal peak structure, likely due to the agglomeration of particles released during the printing process. It is worth noting that the OPS and SMPS operate on slightly different physical principles; thus, there is some degree of error when combining the data. However, the only filament with any notable peak in the OPS range was Brand C green, with a small mass detected at 2.5 microns diameter. Most filaments did not have a notable number of particles detected by OPS; thus, any difficulties in combining these data would not have affected the outcome.

Though the particle formation process for 3D printers has not yet been fully elucidated, it is theorized that 3D printers release primarily ultrafine particles that nucleate and agglomerate into larger particles, as evidenced by scanning electron microscopy (SEM) imaging (Hill et al. 2022). As the printing material undergoes heating and extrusion, VOCs are released as vapors and cool upon exposure to the ambient environment or printing chamber (Ngo et al. 2018). One study has investigated the role of cooled VOCs in particle formation during 3D printing processes (Potter et al. 2019). Their findings demonstrated that certain VOCs commonly used in printing materials, such as styrene and acrylates, exhibit high nucleation potential under cooling conditions typical of 3D printing environments. Since larger particles could be formed partly due to VOCs, the greater quantity of VOCs released by ABS filaments could also be part of why the ABS filaments emitted more particles, in addition to the higher print temperatures used for ABS filaments. ABS filaments have previously been found to release greater mass concentrations and a variety of VOCs than PLA filaments (Davis et al. 2019; Romanowski et al. 2023; Zhang and Black 2023).

Considering the biological responses induced by each filament group, the ABS filaments appear to cause a greater cellular response than the PLA filaments. Other studies also showed a significant response from ABS exposure, including effects on viability, inflammation, and GSH (Fang et al. 2023; Farcas et al. 2022; He et al. 2024; Kim et al. 2022). The biological response induced by exposure to ABS filaments could be correlated with higher emissions in both PM and VOCs. While none of the three metal filaments had a significant GSH value, steel had the lowest average GSH response of all measured filaments. It was narrowly outside of significance (*p* = 0.0867), paired with a 5% average decrease in cell viability, producing a unique cause for concern due to its decrease in both endpoints. Thus, ABS filaments caused a more consistent effect on the cells correlated with overall cell stress. Still, the steel filament seems most likely to cause oxidative stress of the filaments tested in this study. While iron has been shown to be cytotoxic (Eaton and Qian 2002; Fraga and Oteiza 2002; Núñez et al. 2012), other studies examining metal-containing filaments identified steel as less concerning than other metals such as nickel, copper, and titanium (Singh et al. 2021; Vallabani et al. 2022). Ultimately, further studies are needed to probe the concentration of metal particles in emissions from metal-filler-based filaments and the potential toxicity of these metal particles and other filament additives on lung and systemic health (Tedla et al. 2022).

Limitations of this study include the exposure time being restricted to 1 h, which limits the concentration of the test atmosphere exposed to the cells. The particle characterization performed over 60-s sweeps in the BSC also restricted the total number of particles measured. GSH and MTS assays had high errors due to both inherent variation in the cells and inconsistencies in the application of the kit, particularly plate reader temperature. While the TDU temperatures did not perfectly match the 3D printing temperatures, this is a standard method for VOC extraction across a wide range of experimental setups (Emmons et al. 2019; Veenaas et al. 2020; Vu et al. 2018). TDU and 3D printing also vary intrinsically due to the filament only briefly passing through the heated nozzle compared to the 3-min exposure in the TDU.

Future studies should continue to test the wide swathes of filaments available for their effect on overall cell health, both in the 3D printing technique assessed here and in other styles of 3D printing. These studies could also investigate the chemical composition of VOCs and PM produced by each filament. Longer exposure times could help determine the magnitude of risk posed by 3D filaments, and a series of exposure times could be used to determine a dose–response curve. Since 3D printed aerosols are comprised of ultrafine particles, additional studies need to focus on lung cells and explore the effects of these emissions on various tissue and cell types throughout the body. Further studies can probe oxidative stress using alternative assays such as OxyBURST™ Green, Griess reagent, or DTT. Inflammation also seems to have support in the literature as being induced by 3D printing emissions, exemplified by exhaled nitric oxide and IL-5 changes in short-term human studies (Gümperlein et al. 2018; Würzner et al. 2022), and warrants further investigation.

## Conclusion

3D printing has become common across residential, occupational, and scholastic environments. Our results show that ABS filaments produce the most PM and a trend toward the most biological reactivity. Filaments vary within and across material categories; however, steel filaments could pose a unique risk to individuals exposed to 3D printing. General safety practices should be employed to minimize exposure, such as avoiding keeping a household 3D printer in a confined space with low air circulation (*e.g.*, a closet), as well as avoiding spending many consecutive hours near an active printer; for example, users who are accustomed to sleeping next to their active printer. Operations involving ABS filaments may warrant additional caution, such as confining the 3D printer to an enclosed space with an effective extraction system.

## Data Availability

Data will be made available upon request.
